# Role of Rituximab Addition to First-line Chemotherapy Regimens in Nodular Lymphocyte-predominant Hodgkin Lymphoma: A Study by Fondazione Italiana Linfomi

**DOI:** 10.1097/HS9.0000000000000837

**Published:** 2023-04-04

**Authors:** Manuel Gotti, Roberta Sciarra, Alessandro Pulsoni, Francesco Merli, Stefano Luminari, Caterina Zerbi, Livio Trentin, Alessandro Re, Chiara Rusconi, Simonetta Viviani, Andrea Rossi, Federica Cocito, Barbara Botto, Erika Meli, Antonello Pinto, Irene Dogliotti, Guido Gini, Benedetta Puccini, Francesca Ricci, Luca Nassi, Alberto Fabbri, Anna Marina Liberati, Michele Merli, Andrea Riccardo Filippi, Maurizio Bonfichi, Valentina Zoboli, Germana Tartaglia, Giorgia Annechini, Gianna Maria D’Elia, Ilaria Del Giudice, Isabel Alvarez, Andrea Visentin, Stefano Pravato, Daniela Dalceggio, Chiara Pagani, Silvia Ferrari, Caterina Cristinelli, Tanja Lazic, Virginia Valeria Ferretti, Umberto Ricardi, Luca Arcaini

**Affiliations:** 1 Division of Hematology, Fondazione IRCCS Policlinico San Matteo, Pavia, Italy; 2 Department of Molecular Medicine, University of Pavia, Italy; 3 Hematology, Department of Translational and Precision Medicine, Sapienza University of Rome, Italy; 4 Division of Hematology, Azienda USL-IRCCS of Reggio Emilia, Italy; 5 Surgical, Medical and Dental Department of Morphological Sciences related to Transplant, Oncology and Regenerative Medicine, University of Modena and Reggio Emilia, Italy; 6 Hematology Unit, Department of Medicine - DIMED, University of Padova, Italy; 7 Division of Hematology, Spedali Civili, Brescia, Italy; 8 Division of Hematology and Blood Marrow Transplantation, Fondazione IRCCS Istituto Nazionale Tumori, Milan, Italy; 9 Hematology and Bone Marrow Transplant Unit, ASST Papa Giovanni XXIII, Bergamo, Italy; 10 Division of Hematology, ASST Monza, Ospedale S. Gerardo, Monza, Italy; 11 Division of Hematology, Azienda Ospedale Città della Salute e della Scienza, Torino, Italy; 12 Division of Hematology, ASST Grande Ospedale Metropolitano Niguarda, Milano, Italy; 13 Hematology-Oncology and Stem-Cell Transplantation Unit, Department of Hematology and Developmental Therapeutics, Istituto Nazionale Tumori, Fondazione G. Pascale, IRCCS, Napoli, Italy; 14 Division of Hematology, Department of Molecular Biotechnologies and Health Sciences, University of Torino, Italy; 15 Department of Hematology, Ospedali Riuniti, Ancona, Italy; 16 Division of Hematology, Ospedale Careggi, Firenze, Italy; 17 Humanitas Cancer Center, IRCCS Humanitas Research Hospital, Rozzano, Milano, Italy; 18 Hematology, Department of Translational Medicine, AOU Maggiore della Carità and University of Eastern Piedmont, Novara, Italy; 19 Division of Hematology, Azienda Ospedaliero- Universitaria Senese, Siena, Italy; 20 Division of Hematology, Azienda Ospedalaliera S. Maria di terni – Università degli Studi di Perugia, Italy; 21 Division of Hematology, Ospedale di Circolo e Fondazione Macchi, Varese, Italy; 22 Radiation Oncology, Fondazione IRCCS Policlinico San Matteo, Pavia, Italy; 23 Department of Clinical, Surgical, Diagnostic and Pediatric Sciences, University of Pavia, Italy; 24 Service of Clinical Epidemiology and Biometry, Fondazione IRCCS Policlinico San Matteo, Pavia, Italy; 25 Radiation Oncology, Department of Oncology, University of Turin, Italy

## Abstract

Nodular lymphocyte-predominant Hodgkin lymphoma (NLPHL) is a rare entity whose neoplastic cells retain a B-cell phenotype with expression of CD20. Radiotherapy is recommended for favorable stage IA disease while for other stages guidelines suggest therapeutic strategies similar to those used for classic HL. The role of rituximab, although quite widespread, is not completely elucidated. We retrospectively analyzed baseline characteristics of 308 consecutive patients with NLPHL diagnosed in 19 Italian centers from 2000 to 2018. With a median follow-up of 8.4 years (interquartile range: 4.5–12.4) for treated patients, median overall survival (OS) was not reached and estimated 5-year OS was 97.8% and 5-year progression-free survival (PFS) was 84.5%. Five-year cumulative incidence of histological transformation was 1.4%, 95% confidence interval (CI), 0.5%-3.8%. After adjusting for lymphocyte count, splenic involvement, bulky disease and B symptoms (fever, drenching night sweats, unintentional loss >10% of body weight within the preceding 6 months), patients with stage II or more showed superior PFS with immunochemotherapy in comparison to chemotherapy alone (hazard ratio = 0.4, 95% CI, 0.2-0.8; *P* = 0.015). Our data suggest an advantage of the use of rituximab combined with chemotherapy ± radiotherapy in the treatment of stage II–III–IV NLPHL.

## Graphical abstract

**Figure:**
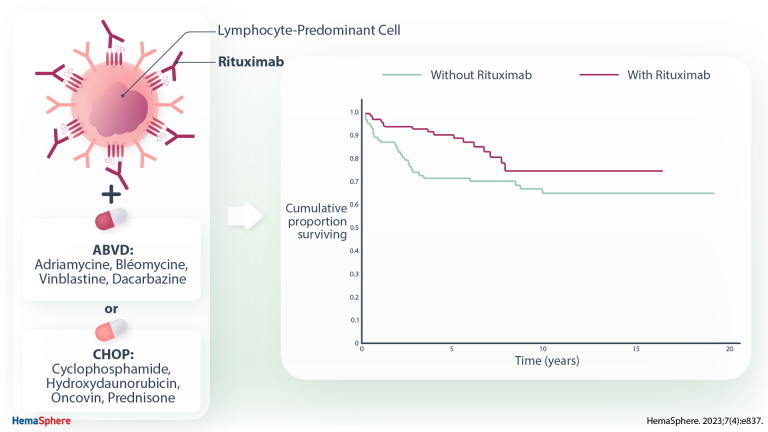

## INTRODUCTION

Nodular lymphocyte-predominant Hodgkin lymphoma (NLPHL) is a rare entity, accounting for approximately 5% of all Hodgkin lymphoma (HL) cases. The histological hallmark of NLPHL is the presence of atypical large malignant cells, known as lymphocyte-predominant cells (“popcorn cells”), staining consistently positive for the B-cell marker CD20 and negative for CD15 and CD30.^1^

NLPHL is often diagnosed in early stage and shows a tendency to relapse multiple times but with a long survival expectancy. Despite its generally indolent course, a subset experiences histological transformation (HT) into aggressive non-Hodgkin lymphoma (NHL).^2–4^ The rate of transformation varies significantly across studies (from nearly 1% to 17%)^2–8^; splenic involvement and tumor size > 5 cm have been reported as risk factor for HT.^9^

European Society Medical Oncology (ESMO) guidelines recommend the use of radiotherapy (RT) alone for stage IA patients without German Hodgkin Study Group (GHSG) clinical risk factors (large mediastinal mass, extranodal lesions, elevated erythrocyte sedimentation rate, and ≥3 nodal areas), whereas National Comprehensive Cancer Network (NCCN) guidelines support the role of RT in stage IA and IIA.^10–12^

Evidence regarding the treatment of advanced cases is less strong and these patients usually receive the same treatment adopted for classical HL.

Commonly used chemotherapy regimens include doxorubicin, bleomycin, vinblastine, and dacarbazine (ABVD) and cyclophosphamide, doxorubicin, vincristine, and prednisone (CHOP). Notably, the expression of CD20 by neoplastic cells provides a rationale for the use of rituximab (R) in NLPHL but its use in combination with chemotherapy has been investigated by only a few studies.^13–15^ These findings assessed the activity and tolerability of rituximab in NLPHL but retrospective series provided conflicting results on the impact of immunochemotherapy on progression-free survival (PFS) and overall survival (OS) in comparison to chemotherapy alone.^13,16–18^

The ESMO guidelines for advanced stage NLPHL recommend classic HL (cHL)–directed chemotherapy, whereas NCCN guidelines suggest either cHL or B-cell NHL-directed chemotherapy regimens, including R-CHOP.^10,11^

Based on these premises, we collected a large series of consecutive cases of NLPHL to identify clinical features and treatment strategies with a focus on the impact of the addition of rituximab to the chemotherapeutic backbone.

## METHODS

### Patient selection

We conducted a multi-institutional retrospective analysis on behalf of the Fondazione Italiana Linfomi (FIL): consecutive NLPHL subjects aged 18 years or older and diagnosed between the years 2000 and 2018 in 19 centers of FIL were enrolled.

Histological diagnosis of NLPHL was established according to the World Health Organization criteria^1^ by expert hemopathologists belonging to the FIL pathologists’ panel.

We collected clinical and laboratory data at diagnosis, management, response evaluation to treatment, and outcome. We registered progression of disease, transformation into high-grade lymphoma or relapse, and long-term toxicities.

Initial staging followed Cotswolds-modified Ann Arbor classification for those patients in whom only computed tomography (CT) scan and bone marrow biopsy were performed, otherwise the Lugano classification of 2014 was applied for those patients staged with fluorodeoxyglucose-positron emission tomography (FDG-PET) scan.^19,20^ Based on the previous study by Xing et al,^9^ bulky disease was defined as a mass of more than 5 cm at diagnosis; splenic involvement was defined as splenomegaly more than 13 cm in vertical length or by the presence of nodules at CT and/or PET-CT evaluation.

Response evaluation was assessed following the 2007 Revised Response Criteria or the 2014 Lugano Classification Criteria, according to the availability of FDG-PET/CT scan at the end of the treatment.^20,21^

The local Ethic Committee of participating Centers approved this study and research was conducted in accordance with the Declaration of Helsinki.

### Statistical analysis

The primary endpoint of the study was to assess the outcome of NLPHL patients in terms of progression-free survival.

Quantitative variables were summarized as median and interquartile range (IQR). Qualitative variables were described as counts and percentages of each category.

PFS was defined as the time since the start of therapy until disease progression, relapse, initiation of further therapy, histologic transformation into B-NHL, or death from any cause. If none of these events had occurred, PFS was censored at the date of the last follow-up. OS was measured from the start of treatment until death from any cause and was censored at the last date of survival status; both OS and PFS were referred only to the cohort of treated patients.

OS and PFS were estimated by Kaplan-Meier product limit method and the comparison of outcome between groups of patients was evaluated via the log-rank test. In patients with stage II/III/IV who received chemotherapy, the effect of treatment with rituximab and baseline characteristics on PFS was tested by univariable proportional hazard Cox model. Clustered sandwich estimator was applied in the standard errors estimation, to allow for intracenter correlation. Variables that in univariable analysis presented with a *P* value lower than 0.1 were inserted in a multivariable model, provided they were not too correlated with each other. Harrell’s *C* index was used to compare predictive power of survival models. *P* values lower than 0.05 were considered significant. Statistical analysis was performed using Stata 16 (StataCorp. 2019. *Stata Statistical Software: Release 16*. College Station, TX: StataCorp LLC).

## RESULTS

### Baseline clinical characteristics and treatment details

Baseline characteristics of 308 patients are summarized in Table 1. Median age at diagnosis was 44 years (IQR: 33–57) with male predominance (70%). Disease was in stage I in 29% of patients and in stage II in 41% while only one-third of patients presented with stage III–IV disease (19% stage III and 11% stage IV). Bulky disease was detected in 20 patients (7%). Extranodal disease was infrequent (11%): skeleton, lung, liver, pharynx, muscle tissue, subcutaneous tissue, pancreas, and bowel; 10 cases had more than 1 extranodal site involvement. Bone marrow involvement was quite rare (1%): splenic involvement was identified in 35 patients (11%).

**Table 1:** Baseline Features of 308 Patients With Nodular Lymphocyte-predominant Hodgkin Lymphoma

Feature	N. (%)
Age (y)	
Median	44
Range, IQR	33–57
≤45 y	162
Male/female	216/92 (70/30)
Stage	
I	89 (29)
II	127 (41)
III	59 (19)
IV	33 (11)
B symptoms	27/306 (9)
Extranodal disease	35/308 (11)
Spleen involvement	35/308 (11)
Bone marrow involvement	4/305 (1)
Hemoglobin <10.5 g/dL	7/279 (3)
Lymphocytes <8%	4/272 (1)
Albumin <4 g/dL	40/235 (17)
LDH > UNL	32/261 (12)
Bulky mass (>5 cm)	20/303 (7)
Elevated ESR^a^	16/225 (7)
β_2_-microglobuline > UNL	32/186 (17)

a ≥50 mm/h if not B symptoms; ≥30 if B symptoms.

ESR = erythrocyte sedimentation rate; IQR = interquartile range; LDH = lactate dehydrogenase; UNL = upper normal limit.

Overall, only 14 patients (4%) were managed with a watch and wait approach (9%, 4%, 2%, and 0% among stages I, II, III, and IV, respectively), defined as the intention not to treat and no treatment administration within 6 months from diagnosis. Treatment details are provided in Table 2. Median time from diagnosis to treatment was 1.4 months (IQR: 0.9–2.3).

**Table 2:** Details of Treatment Adopted for 294 Patients With Nodular Lymphocyte-predominant Hodgkin Lymphoma Who Have Undergone a Therapeutic Regimen According to Disease Stage

	Stage I (n = 81)	Stage II–III–IV (n = 213)	Total (n = 294)
**No rituximab**	**63 (77.8%**)	**90 (42.3%**)	**153 (52.0%**)
* *RT alone	29 (35.8%)	9 (4.2%)	38 (12.9%)
* *Chemotherapy alone	6 (7.4%)	31 (14.6%)	37 (12.6%)
ABVD alone	6	30	36
CHOP alone	0	1	1
* *Chemotherapy + RT	28 (34.5%)	50 (23.5%)	78 (26.5%)
ABVD + RT	28	50	78
CHOP + RT	0	0	0
**With rituximab**	**18 (22.2%**)	**123 (57.7%**)	**141 (48.0%**)
R alone	3 (3.7%)	8 (3.7%)	11 (3.8%)
R + RT	2 (2.5%)	3 (1.4%)	5 (1.7%)
R-chemotherapy	2 (2.5%)	80 (37.6%)	82 (27.9%)
R-ABVD	2	41	43
R-CHOP	0	39	39
R-chemotherapy + RT	11 (13.6%)	32 (15%)	43 (14.6%)
R-ABVD + RT	9	25	34
R-CHOP + RT	2	7	9

ABVD = doxorubicin, bleomycin, vinblastine, and dacarbazine; CHOP = cyclophosphamide, doxorubicin, vincristine, and prednisone; R = rituximab; RT = radiotherapy.

Eighty-six percent of treated patients presenting with stage I (n = 70) received RT alone or combined to chemo- and immunochemotherapy. Ninety-one percent of patients with stage II/III/IV (n = 193) were treated with chemotherapy, combined with RT in 38%.

Of 193 patients in stage II/III/IV treated with chemotherapy, 112 (58%) patients received rituximab as part of induction; among these 112 patients, 66 (59%) received R-ABVD and 46 (41%) R-CHOP. Almost all patients treated without rituximab received ABVD or ABVD-like regimen (80/81, 99%).

### Outcome and impact of rituximab

Response to treatment was evaluable in 294 intentionally treated patients; patients managed with a watch and wait approach were excluded from this analysis (details of responses are reported in Suppl. Tables S1 and S2).

At a median follow-up of 8.4 years (IQR: 4.5–12.4) for treated patients, median OS was not reached and estimated 5-year OS (95% confidence interval [CI]) was 97.8% (95.1%–99.0%) and 5-year PFS (95% CI) was 84.5% (79.7%–88.3%).

**Figure 1.**
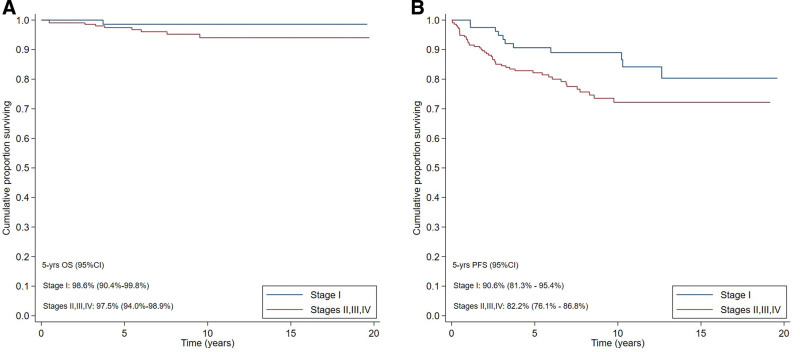
**Survival outcome.** OS (A) and PFS (B) of 294 patients with nodular lymphocyte-predominant Hodgkin lymphoma according to stage. CI = confidence interval; OS = overall survival; PFS = progression-free survival.

In stage I NLPHL, 5-year OS was 98.6% and 5-year PFS was 90.6%. In stage II–III–IV NLPHL, 5-year OS was 97.5% and 5-year PFS was 82.2% (Figure 1).

Among 10 reported deaths, none was due to lymphoma; we registered 5 secondary cancers (1 prostatic, 2 gastric, 1 nasopharyngeal, and 1 acute myeloid leukemia) in our cohort occurring after a median time of 5 years from diagnosis (range 3–7 years).

In 193 stage II, III, and IV patients treated with a chemotherapy-based approach (only for 1 patient, the response data were not available), the median follow-up time for the immunochemotherapy cohort was 73 months (IQR: 44–103), whereas for patients treated without rituximab, the median follow-up time was 134 months (IQR: 104–169). This difference is due to the progressive introduction of rituximab in first-line regimens. In particular, we observed a gradual increase in the incidence of patients treated with immunochemotherapy over the years: 1 of 18 (5.6%) between 2000 and 2005; 21 of 58 (36.2%) between 2005 and 2010; 42 of 63 (66.7%) between 2010 and 2015; 48 of 54 (88.9%) between 2015 and 2020.

**Figure 2.**
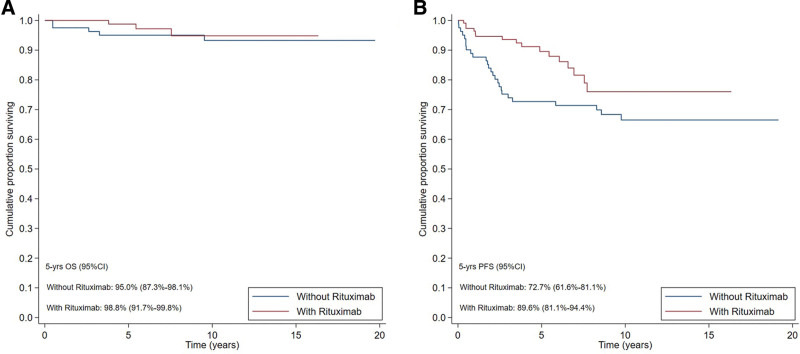
**Survival outcome.** OS (A) and PFS (B) of 193 chemotreated patients with stage II, III, IV nodular lymphocyte-predominant Hodgkin lymphoma according to the use of rituximab. CI = confidence interval; OS = overall survival; PFS = progression-free survival.

The 5-year PFS was 89.6% in the former group and 72.7% in the latter (*P* = 0.034), while no difference was found in terms of OS (*P* = 0.509) (Figure 2). In an explorative analysis limited to patients treated with immunochemotherapy, no significant difference was found in terms of OS and PFS according to the adopted chemotherapy regimen (*P* = 0.198 and *P* = 0.477, respectively). Further analysis is available in supplemental digital content and reports the outcome in terms of OS and PFS distinguishing early stage (II) versus advanced stage (III–IV).

In univariate analysis conducted only in patients with stages II–IV treated with chemotherapy, the no-use of rituximab, splenic involvement, Ann Arbor stage III and IV, lymphopenia (<8%), presence of B symptoms and bulky disease were associated to a shorter PFS. In multivariate analysis, no-use of rituximab, presence of bulky disease, and splenic involvement were associated to a worse PFS (Table 3). At multivariate analysis, we excluded stage as a variable (III–IV versus II) because it was closely related to spleen involvement. An alternative model with stage instead of spleen involvement was carried out but this model presented a lower Harrel’s C (0.71 versus 0.73).

**Table 3:** Crude (Univariate Analysis) and Adjusted (Multivariate Model) Effect of Rituximab and Baseline Characteristics on Progression-free Survival in 193 Patients With Stage II/III/IV Nodular Lymphocyte-predominant Hodgkin Lymphoma Who Received Chemotherapy

				Univariable Analysis		Multivariable Model	
	CHEMO (n = 81)	R-CHEMO (n = 112)	*P* value	HR (95% CI)	*P* Value	HR (95% CI)	*P* Value
Age at diagnosis (y), median (IQR)	42 (35–53)	49 (39–59)	0.014	1.0 (1.0-1.0)	0.915		-
Stage, n (%)			0.028				
II	53 (65.4%)	55 (49.1%)		Ref	Ref	-	-
III–IV	28 (34.6%)	57 (50.9%)		2.0 (1.1-3.6)	0.030	-	-
Hemoglobin (g/dL), median (IQR)	14.4 (13.2–15.4)	14.3 (13.1–15.2)	0.648	0.9 (0.7-1.1)	0.217	-	-
Lymphocytes, n (%)			>0.90				
≥8%	73 (98.7%)	100 (98.0%)		Ref	Ref	Ref	Ref
<8%	1 (1.3%)	2 (2.0%)		4.5 (1.1-18.7)	0.041	2.8 (0.6-13.3)	0.184
Spleen involvement, n (%)			0.239				
No	71 (87.7%)	90 (80.4%)		Ref	Ref	Ref	Ref
Yes	10 (12.3%)	22 (19.6%)		3.9 (2.0-7.6)	<0.001	3.2 (1.4-7.6)	0.007
Bulky, n (%)			0.386				
No	72 (94.7%)	80 (90.9%)		Ref	Ref	Ref	Ref
Yes	4 (5.3%)	8 (9.1%)		2.5 (0.9-7.1)	0.085	3.4 (1.1-10.7)	0.034
B symptoms, n (%)			0.193				
No	74 (91.4%)	95 (84.8%)		Ref	Ref	Ref	Ref
Yes	7 (8.6%)	17 (15.2%)		2.2 (1.1-4.6)	0.034	1.9 (0.8-4.7)	0.155
Albumin, n (%)			0.411				-
≥4 g/dL	56 (84.9%)	72 (78.3%)		Ref	Ref	-	-
<4 g/dL	10 (15.1%)	20 (21.7%)		0.9 (0.3-2.3)	0.804	-	-
Rituximab, n (%)							
No	-	-		Ref	Ref	Ref	Ref
Yes	-	-		0.5 (0.3-1.0)	0.037	0.4 (0.2-0.8)	0.015

CI = confidence interval; HR = hazard ratio; IQR = interquartile range.

### Relapse and HT

Forty-seven patients experienced at least 1 relapse during follow-up; 38 patients relapsed as NLPHL (78%) and 5 patients underwent HT to diffuse large B-cell lymphoma (12%). Median time to HT was 25.1 months (IQR: 20.2–31.8) after primary management, and 5-year cumulative incidence of HT was 1.4% (95% CI: 0.5-3.8). Two of the 5 patients who experienced HT initially presented in stage IIA as NLPHL and both received ABVD and RT as first-line treatment; they showed PD at the final evaluation and, after a rebiopsy showing DLBCL transformation, they were both treated with rituximab, dexamethasone, cytarabine, and cisplatin (R-DHAP) and autologous stem cell transplantation (ASCT), achieving a complete response (CR). One patient was diagnosed with stage IIIA NLPHL, received 6 cycles of R-ABVD obtaining a CR, relapsed as DLBCL with infradiaphragmatic nodal diseases and was successfully treated with bendamustine and rituximab. The other 2 HT cases initially presented in stage IVA NLPHL: the former received only rituximab as first-line therapy with a final CR, relapsed in stage III and was treated with R-CHOP regimen, obtaining a stable CR; the latter was initially treated with combined modality therapy (R-CHOP and RT), showing PD and was subsequently treated with immunochemotherapy and ASCT.

First relapse as NLPHL occurred after a median time of 27.6 months (IQR: 10.7–70.2 months) from the start of therapy. Management at first relapse was heterogeneous; in 16 cases consolidation with autologous stem cell transplantation (SCT) was performed; 1 patient with multiple relapses underwent allogeneic SCT. All patients who underwent autologous SCT obtained a complete remission confirmed at last follow-up.

Among the 22 patients who relapsed as NLPHL and did not received consolidation with ASCT, we observed that all of them, irrespectively of their onset stage and first-line regimen, received rituximab as salvage treatment (alone or combined to chemotherapy or RT) and obtained a durable CR. One patient experienced a second relapse and was lost to follow-up after refusing ASCT; 1 patient died of pharyngeal cancer.

## DISCUSSION

In the present study, we report clinical features, treatment, and outcome of patients with NLPHL diagnosed in 19 centers belonging to the Italian lymphoma cooperative group FIL; we decided to start collection from 2000 due to the implementation of a homogenous characterization of the disease by the panel of FIL pathologists and due to the availability of rituximab.

Population characteristics are superimposable with previous studies: higher incidence in males, a median age at onset higher than that of cHL, an early stage in many cases, a rare extranodal or bone marrow involvement, a low percentage of patients with systemic symptoms, and a sporadic presentation with bulky disease.^16,18,22,23^

Regarding treatment, NLPHL management is well defined only for stage IA. Our study stems from the lack of consensus on the best therapeutic strategy for stages II, III, and IV. ESMO guidelines recommend treating these patients with the same approach as with cHL. However, the advantage offered by the introduction of rituximab in association with chemotherapy is currently still under debate.^8,10,15,24^

In reference to a cHL oriented strategy, a recent retrospective study published by GHSG showed a good outcome in patients with NLPHL treated as cHL (from HD7 to HD15) with 10-year PFS and 10-year OS of around 75% and 92%.^23^ Overall, these data seem to be in line with our results that show a 5-year PFS of around 73% and a 5-year OS of 97% in patients undergoing chemotherapy alone or combined treatment with chemotherapy and RT.

Regarding the role of rituximab combined with chemotherapy, feasibility and efficacy of this approach has been previously described. Fanale et al^13^ reported an excellent outcome in 27 patients with NLPHL treated with R-CHOP (5-year PFS 88.5% and 5-year OS of 100%) and Mocikova et al^15^ reported a 5-year PFS of 90% and a 5-year OS of 100% in 23 patients treated with immunochemotherapy.

The lack of consensus for patients with stage disease other than IA^13,25–27^ prompted us to consider the impact of rituximab in the wide subgroup of patients with stage II–III and IV from our series.

Our data show a significantly better PFS in patients with stage II or higher, who underwent treatment with rituximab, after adjusting for clinical parameters.

Considering that patients receiving chemotherapy without rituximab had ABVD or ABVD-like regimens in the majority of cases, we cannot draw final conclusions on the role of rituximab in this setting, it is noteworthy that patients with advanced NLPHL receiving the intensive BEACOPP regimen do not appear to benefit from the addition of rituximab at least in case of PET-2 positivity.^28^

In comparison with other prognostic parameters previously reported in the NLPHL,^9,29,30^ albeit with the limit of the lack of characterization of variant patterns by Fan classification, we did not find correlation between PFS and albumin, gender, and hemoglobin level.

In addition, we took the opportunity to analyze the role of R-ABVD already described in previous studies with a limited number of cases. The first experience was based on 6 patients and concluded that R-ABVD is less toxic than R-CHOP and reported an estimated 6-year PFS of 75% and OS of 100%.^14^ A second study, based on 24 patients, confirmed the safety of this regimen but suggested to avoid the use of bleomycin in elderly patients as it reported a worse outcome with a 5-year PFS of 80% and a 5-year OS of 100%.^31^ Interestingly, in our series, outcome in terms of PFS and OS after R-ABVD and R-CHOP appears to be similar. Moreover, adjusting for stage (stage III–IV versus II) did not show any significant difference in terms of PFS between the 2 treatment groups (R-CHOP versus R-ABVD; *P* = 0.303). Unfortunately, the small number of events did not allow us to adjust this effect for clinical parameters.

A key point in NLPHL treatment is to delay relapse and to avoid or minimize HT: in our series, we observed a relapse in 17% of patients and most of them relapsed as NLPHL. In the study by Fanale et al,^13^ 9 of 59 patients relapsed (15%), histology was still NLPHL in 7 patients, 2 patients showed HT, and no patient treated with R-CHOP transformed.

In our study, we observed a low rate of HT into DLBCL (5 cases with a 5-year cumulative incidence of HT of around 1.4%).

The risk of HT varies across different studies. A French registry of NLPHL patients, followed for nearly 10 years, indicated a cumulative HT risk of 12%, with a median time to transformation of 4.7 years from NLPHL diagnosis,^3^ while British Columbia Cancer Agency observed a 14% risk of transformation at a median follow-up of 8 years and none of the transformed cases received rituximab.^2^ Finally, the Mayo Clinic reported 220 NLPHL patients with a median follow-up of 16 years and described a transformation rate of 7.7%, with a median time to transformation of 35 months.^6^ Our data do not allow a detailed analysis on refractory/relapsed disease due to the heterogeneity of second-line approach. This limit is described in other papers focused on this theme due to a lack of consensus on the role of autologous SCT or other regimens.^32,33^

To acknowledge the limitations of our study, we underline its retrospective design and a consequent inhomogeneity of follow-up times across the adopted treatments. Furthermore, the lack of formal histological revision prevented the analysis of histological patterns.

In conclusion, our data demonstrate an advantage in terms of outcome by the addition of rituximab to the standard therapeutic strategy (chemotherapy ± RT) in patients with stage II or higher. The introduction of rituximab appears to improve outcome in terms of progression, irrespective of the associated chemotherapeutic regimen. Indeed, no significant differences in terms of OS and PFS were observed between ABVD and CHOP regimens in association to rituximab. Both these approaches can be considered as equally valuable alternatives for treatment of patients with NLPHL, while ABVD alone showed a poorer outcome when administered alone.^9^

Despite the rarity of the disease and the widespread use of rituximab, prospective studies in NLPHL are eagerly awaited to clarify the role of anti-CD20 therapy in this peculiar entity.

## AUTHOR CONTRIBUTIONS

MG conceived and designed the study; AlP, FM, SL, CZ, LT, AlR, CR, AnR, FC, BB, EM, AnP, ID, GG, BP, FR, LN, AF, AML, MM, ARF, MB, VZ, GT, GA, GMD, IDG, IA, AV, SP, DD, CP, SV, SF, CC, VVF, UR; VVF analyzed data; VVF, RS, and CZ created the tables and figures; MG, RS, CZ, and LA wrote the first draft of the manuscript; all the authors critically reviewed and approved the final manuscript.

## DISCLOSURES

LA received advisory honoraria from Roche, Celgene, Janssen-Cilag, Verastem, Eusa Pharma, and Incyte, research support from Gilead, and travel expenses from Roche, Celgene, Janssen-Cilag, and Eusa Pharma, speakers bureau from Novartis. AP declares honoraria from Roche. All the other authors have no conflicts of interest to disclose.

## SOURCES OF FUNDING

This work was supported by the Italian Ministry of Education, University and Research (MIUR) to the Departments of Molecular Medicine (DMM) of the University of Pavia under the initiative “Dipartimenti di Eccellenza (2018–2022)” and by grants of the Fondazione Regionale Ricerca Biomedica (FRRB), Milan, Italy (FRRB project no. 2015-0042, genomic profiling of rare hematologic malignancies, development of personalized medicine strategies, and their implementation into the Rete Ematologica Lombarda clinical network).

## Supplementary Material


