# Supplementary material for: Role of Rituximab Addition to First-line Chemotherapy Regimens in Nodular Lymphocyte-predominant Hodgkin Lymphoma: A Study by Fondazione Italiana Linfomi
Source: Hemasphere. 2023 Apr 4;7(4):e837. doi: 10.1097/HS9.0000000000000837 (PMC10079338; doi:10.1097/HS9.0000000000000837)
Supplement: Supplementary file 1 [file hs9-7-e837-s001.docx]

**Supplemental Table 1**. Response rates according to stage in 294 patients with nodular lymphocyte-predominant Hodgkin lymphoma who received chemotherapy.

|  | **Stage I**  **(n = 81)** | **Stages ≥ II**  **(n = 213)** | p-value |
| --- | --- | --- | --- |
| CR  PR  NR/PD/SD | 81/81 (100%)  0  0 | 192 (90.1%)  13 (6.1%)  8 (3.8%) | 0.008 |

**Supplemental Table 2**. Response rates according to therapeutic regimen in 213 patients with nodular lymphocyte-predominant Hodgkin lymphoma with stage ≥II.

|  | **Response rate in STAGE ≥ II patients**  **(N=213)** | | |
| --- | --- | --- | --- |
|  | **CR** | **PR** | **SD/NR/PD** |
| **Chemo-containing regimen** | 175 (90.7%) | 11 (5.7%) | 7 (3.6%) |
| *ABVD* | 23 (76.7%) | 2 (6.7%) | 5 (16.7%) |
| *CHOP* | 1 (100%) | 0 (0.0%) | 0 (0.0%) |
| *R-ABVD* | 37 (90.2%) | 4 (9.8%) | 0 (0.0%) |
| *R-CHOP* | 38 (97.4%) | 1 (2.6%) | 0 (0.0%) |
| *ABVD + RT* | 47 (94.0%) | 1 (2.0%) | 2 (4.0%) |
| *CHOP+ RT* | - | - | - |
| *R-ABVD+RT* | 25 (100%) | 0 (0.0%) | 0 (0.0%) |
| *R-CHOP+RT* | 4 (57.1%) | 3 (42.9%) | 0 (0.0%) |
| **RT alone** | 9 (100%) | 0 (0.0%) | 0 (0.0%) |
| **R alone** | 6 (75%) | 1 (12.5%) | 1 (12.5%) |
| **RT + R** | 2 (66.7%) | 1 (33.3%) | 0 (0.0%) |

RT: radiotherapy; R: rituximab
