# Supplementary material for: Role of Rituximab Addition to First-line Chemotherapy Regimens in Nodular Lymphocyte-predominant Hodgkin Lymphoma: A Study by Fondazione Italiana Linfomi
Source: Hemasphere. 2023 Apr 4;7(4):e837. doi: 10.1097/HS9.0000000000000837 (PMC10079338; doi:10.1097/HS9.0000000000000837)
Supplement: Supplementary file 2 [file hs9-7-e837-s002.docx]

**SUPPLEMENTAL DIGITAL CONTENT**

**Overall survival (A) and Progression Free Survival (B) in 193 patients with stage II/III/IV nodular lymphocyte-predominant Hodgkin lymphoma who received chemotherapy according to stage and Rituximab.**


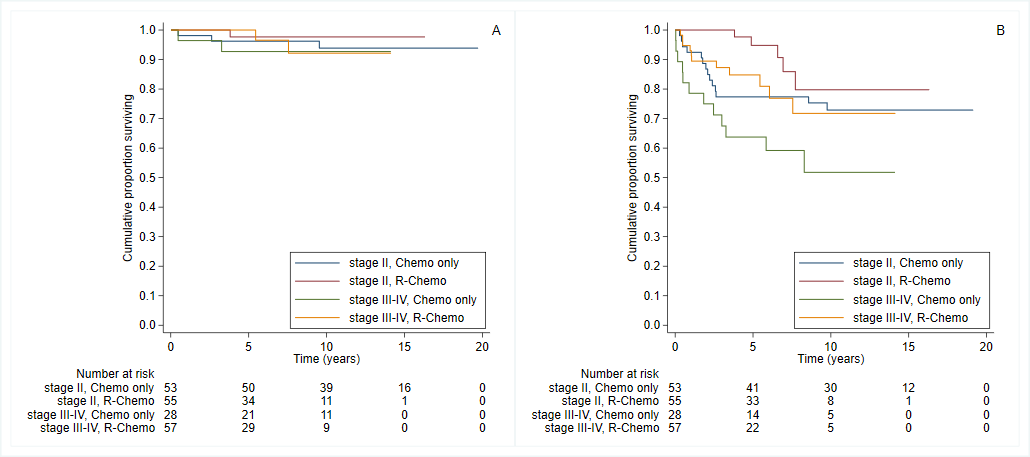


| **III-IV vs II** | **5-yrs OS (95%CI)** | | **5-yrs PFS** | |
| --- | --- | --- | --- | --- |
| R-Chemo | **II**: 97.7% (84.6-99.7) | p=0.542 | **II**: 94.8% (80.6-98.7) | p=0.066 |
|  | **III-IV**: 100% |  | **III-IV**: 84.8% (71.6-92.2) |  |
| Chemo only | **II**: 96.2% (85.7-99.0) | p=0.702 | **II**: 77.4% (63.6-86.5) | p=0.067 |
|  | **III-IV**: 92.7% (73.9-98.1) |  | **III-IV**: 63.8% (43.1-78.6) |  |
